# Photogenerated Carrier Transport Properties in Silicon Photovoltaics

**DOI:** 10.1038/s41598-019-55173-z

**Published:** 2019-12-12

**Authors:** Prakash Uprety, Indra Subedi, Maxwell M. Junda, Robert W. Collins, Nikolas J. Podraza

**Affiliations:** Wright Center for Photovoltaics Innovation and Commercialization & Department of Physics and Astronomy, University of Toledo, Toledo, OH 43606 USA

**Keywords:** Energy science and technology, Materials science, Optics and photonics

## Abstract

Electrical transport parameters for active layers in silicon (Si) wafer solar cells are determined from free carrier optical absorption using non-contacting optical Hall effect measurements. Majority carrier transport parameters [carrier concentration (*N*), mobility (*μ*), and conductivity effective mass (m*)] are determined for both the n-type emitter and p-type bulk wafer Si of an industrially produced aluminum back surface field (Al-BSF) photovoltaic device. From measurements under 0 and ±1.48 T external magnetic fields and nominally “dark” conditions, the following respective [n, p]-type Si parameters are obtained: *N* = [(3.6 ± 0.1) × 10^18^ cm^−3^, (7.6 ± 0.1) × 10^15^ cm^−3^]; *μ* = [166 ± 6 cm^2^/Vs, 532 ± 12 cm^2^/Vs]; and m* = [(0.28 ± 0.03) × *m*_*e*_, (0.36 ± 0.02) × *m*_*e*_]. All values are within expectations for this device design. Contributions from photogenerated carriers in both regions of the p-n junction are obtained from measurements of the solar cell under “light” 1 sun illumination (AM1.5 solar irradiance spectrum). From analysis of combined dark and light optical Hall effect measurements, photogenerated minority carrier transport parameters [minority carrier concentration (*Δp* or *Δn*) and minority carrier mobility (*μ*_*h*_ or *μ*_*e*_)] under 1 sun illumination for both n- and p-type Si components of the solar cell are determined. Photogenerated minority carrier concentrations are [(7.8 ± 0.2) × 10^16^ cm^−3^, (2.2 ± 0.2) × 10^14^ cm^−3^], and minority carrier mobilities are [331 ± 191 cm^2^/Vs, 766 ± 331 cm^2^/Vs], for the [n, p]-type Si, respectively, values that are within expectations from literature. Using the dark majority carrier concentration and the effective equilibrium minority carrier concentration under 1 sun illumination, minority carrier effective lifetime and diffusion length are calculated in the n-type emitter and p-type wafer Si with the results also being consistent with literature. Solar cell device performance parameters including photovoltaic device efficiency, open circuit voltage, fill factor, and short circuit current density are also calculated from these transport parameters obtained via optical Hall effect using the diode equation and PC1D solar cell simulations. The calculated device performance parameters are found to be consistent with direct current-voltage measurement demonstrating the validity of this technique for electrical transport property measurements of the semiconducting layers in complete Si solar cells. To the best of our knowledge, this is the first method that enables determination of both minority and majority carrier transport parameters in both active layers of the p-n junction in a complete solar cell.

## Introduction

Minority carrier transport parameters critically affect operation and performance of many p-n junction semiconductor devices including bipolar transistors and solar cells^1–4^. Knowledge of intrinsic carrier concentration, effective equilibrium minority carrier concentration, minority carrier mobility, and effective carrier lifetime obtained by theoretical and experimental methods are essential parameters for modeling semiconductor devices to understand device physics and to optimize performance^1–6^. For solar cells in particular, both open circuit voltage (*V*_*oc*_) and photogenerated current density (*J*_*L*_) depend on minority carrier mobility and effective carrier lifetime in the absorber material^7^.

Silicon (Si) wafer photovoltaic (PV) devices are currently the most mature and dominant technology in the solar module market accounting for ~90% of total global production^8^. As such, some of the pertinent minority carrier transport properties of Si have been extracted using photoluminescence^9,10^, a combination of steady state electrical and transient optical techniques^5^, photoconductance decay^6,11^, analysis of frequency domain transient photocurrent^12^, theoretical modeling^13^, *V*_*oc*_ decay^14^, quasi-steady-state photoconductance and quasi-steady-state *V*_*oc*_^7^, resonant-coupled photoconductive decay^15^, and a combination of transient and quasi-steady-state photoconductance^16^. Dziewior *et al*. have measured the minority carrier mobilities in heavily doped n- and p-type Si^9^. Swirhun *et al*. found the minority carrier hole mobility in heavily doped n-type Si is about double that of the majority carrier hole mobility in p-type Si doped to a similar carrier concentration^10^. Wang *et al*. found that the minority hole mobility in n-type Si is approximately double than that of the majority hole mobility in p-type Si^12^. Law *et al*. studied the variation of minority carrier mobility as a function of dopant concentration using theoretical modeling^13^. Swirhun *et al*. have reported the variation of minority carrier mobility in p-type Si as a function of dopant concentration and found that the electron mobility in p-type Si is about 2.5 times that of the electron mobility in n-type Si doped to a similar carrier concentration^5^. Sproul *et al*. have reported the variation of the minority carrier mobility in n-type and p-type Si as a function of dopant concentration^6^. The variation of minority carrier mobility with respect to temperature in the bulk layer of a Si wafer solar cell has been reported using *V*_*oc*_ decay^14^. Neuhaus *et al*. have studied the injection level dependence of the sum of minority and majority carrier mobilities in the bulk layer of Si wafer solar cell^7^. Even with all this extensive background of Si electrical transport studies, however, extraction of both minority and majority carrier properties in both the p- and n-type components of a Si solar cell p-n junction is not straightforward. In this work optical Hall effect measurements of a Si wafer solar cell under nominally dark and 1 sun illumination conditions are used to determine both majority and minority carrier transport properties.

**Figure 1:**
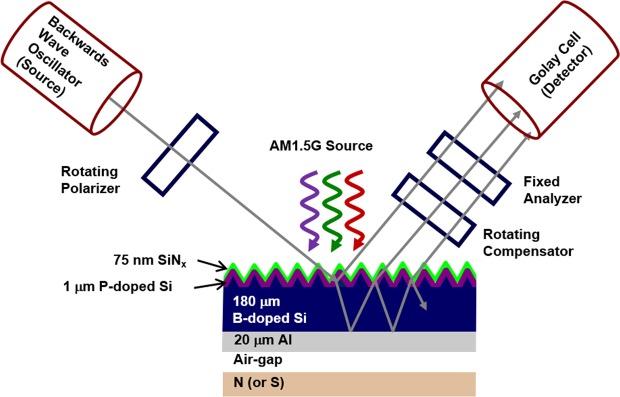
Schematic of a Si wafer aluminum back surface field (Al-BSF) solar cell mounted on a magnet for optical Hall effect measurements based on terahertz (THz) range spectroscopic ellipsometry under 1 sun illumination (AM 1.5 solar irradiance spectrum)^1,3,15^. More detailed information regarding this type of experimental setup can be found in ref. ^30^.

In general, complete Si solar cells are a multiple layer stack of p- and n-type semiconductors along with electrical contact and passivation layers. A typical Si wafer solar cell has a p-type base with the near-surface (top 1 μm) more heavily doped with a pentavalent impurity yielding the emitter. Aluminum back surface field (Al-BSF) solar cells are the most common solar cells. Direct electrical measurement of transport parameters for individual semiconductors in these device structures is often difficult or impossible. Optical Hall effect via magnetic field dependent frequency domain terahertz (THz) range spectroscopic ellipsometry is a potential alternative method that enables extraction of majority carrier transport parameters such as conductivity effective mass (m*), carrier concentration (*N*), and mobility (*μ*) of individual layers even in the complex multilayer structures, but without making physical contact^17–24^. Figure 1 shows a schematic illustration depicting a THz range spectroscopic ellipsometer used for the analysis of a Si wafer Al-BSF solar cell with an adjustable magnetic field dependence (0, ±1.48 T) under 1 sun illumination (AM1.5 solar irradiance). Majority carrier transport properties have also been studied previously using long wavelength range spectroscopic ellipsometry with results in good agreement with direct electrical measurements^25–29^. In comparison to long wavelength range ellipsometric measurements collected without an external magnetic field, the optical Hall effect measurements described here provide the capability of determining an increased number of transport parameters simultaneously and with increased accuracy^30^. For instance, in a previous study without the external magnetic field, *N* or *μ* has been obtained optically for cadmium telluride (CdTe) when the other value is known and both *N* and *μ* for Si wafers have been obtained^29^. With magnetic field dependent measurements, both *N* and *μ* for thin film CdTe and all three of *N*, *μ*, and m* for Si wafers have been successfully measured^30^.

Optical Hall effect measurements are now adapted to deduce minority electrical transport properties [minority carrier mobility (*μ*_*h*_ or *μ*_*e*_) and equilibrium photogenerated minority carrier concentration (*Δp* or *Δn*)] and majority carrier *N*, *μ*, and m* of active layers within a complete solar cell. In commercially produced Al-BSF Si wafer solar cells, an aluminum metal layer is commonly used as the rear-side electrical contact to the p-type wafer. The junction is formed from an n-type emitter on the p-type wafer base with a silicon nitride (SiN_x_) antireflection coating and screen-printed silver front and aluminum rear contacts^31^. The cell surface is pyramidally textured to enhance absorption by light trapping, while the SiN_x_ dielectric coating serves to passivate dangling bonds present on the Si surface, which then reduces recombination centers for photogenerated carriers to enhance solar cell performance^32^. Here majority carrier *N*, *μ*, and m* are determined for both the n-type emitter and p-type wafer Si regions of an industrially produced Al-BSF Si solar cell from THz spectral range optical Hall effect measurements performed in nominally “dark” conditions. Contributions from photogenerated carriers in both the p- and n-type regions are obtained from optical Hall effect measurements of the solar cell under “light” 1 sun illumination (AM 1.5 solar irradiance spectrum). Analysis of the dark and light optical Hall effect measurements yields minority and majority transport characteristics for each Si component of the p-n junction within the complete solar cell. The transport properties are then used to calculate minority carrier lifetime and diffusion length of each layer as well as the PV device performance parameters.

## Experimental Details

A commercially fabricated 15.6 × 15.6 cm^2^ pseudo-square Al-BSF Si wafer solar cell (CNH Industrial) has been purchased and characterized. The basic Al-BSF design consists of a 1 μm thick n-type phosphorus doped Si emitter, a 180 μm thick p-type boron doped Si wafer, a 75 nm SiN_x_ antireflection coating, three screen printed 1 × 154 mm^2^ silver bus bar front contacts, and nine silver 2 × 22 mm^2^ bus bars soldered to a 20 μm thick aluminum layer back contact^31,32^. Finger bars on the front surface are 0.1 mm wide and separated by 1.4 mm. The front surface is pyramidally textured. PV device performance parameters including the short circuit current density (*J*_*sc*_), open circuit voltage (*V*_*oc*_), fill factor (*FF*), and efficiency (*η*) are determined from current-voltage (*J-V*) measurements collected under 1 sun illumination. Experimental error on solar cell performance parameters from *J-V* are determined from one standard deviation of the measurement.

Mueller matrix spectra are collected *ex-situ* at 50, 60, and 70° angles of incidence in the THz spectral range from 3 to 34 cm^−1^ (3.3 mm to 290 μm) for the Si wafer solar cell (J. A. Woollam Co., THz-VASE)^33^. Eleven Mueller matrix elements normalized to M_11_ (m_12_, m_13_, m_21_, m_22_, m_23_, m_31_, m_32_, m_33_, m_41_, m_42_, and m_43_) are obtained. Measured data are collected from an area of the cell oriented to avoid the front silver bus bars. The configuration of the ellipsometer used here differs from that described by Hofmann *et al*.^33^ in the use of a rotating polarizer before interaction with the sample and a rotating compensator followed by fixed analyzer after interaction with the sample. For each case, sets of eleven Mueller matrix element spectra at the three angles are collected with the sample under applied magnetic fields of 0 and ±1.48 T. A Ni-Cu-Ni coated Neodymium N42 permanent magnet 3″ × 3″ × 1/2″ in size generates a maximum magnetic field of ±1.48 T. The magnet is mounted on the sample stage goniometer with double-sided tape on the corners, and then the sample is directly mounted on the magnet with known north or south (N or S) polarity. After the data are collected with one polarity, the magnet is removed and remounted to reverse the polarity. The separation between the sample and the magnet introduces an air-gap into the optical-structural model. Similar optical Hall effect measurements are also performed under ~1 sun illumination (AM 1.5 solar irradiance spectrum) generated by a fiber optic illuminator (Dolan-Jenner, Fiber-Lite Model 190).

For these optical Hall effect measurements, THz range spectroscopic ellipsometry is used as the optical probe. All three sets of eleven Mueller matrix elements spectra are analyzed simultaneously in a model sensitive to the complex dielectric function (*ε* = *ε*_1_ + i*ε*_2_) spectra as well as to wafer thickness and air gap thickness when applicable. The contributions to the optical response in this spectral range include those due to free carrier absorption, described classically by the Drude model^34^. The Drude model yields a complex dielectric function term defined by:

1 $${\varepsilon }_{Drude}(\omega )=\frac{-{q}^{2}N\mu }{{\varepsilon }_{0}({m}^{\ast }\mu {\omega }^{2}+iq\omega )}$$

where *q*, *N*, *μ, ε*_0_, and m* are the elementary charge, carrier concentration, mobility, vacuum permittivity, and carrier conductivity effective mass, respectively. Resistivity (*ρ*) and scattering time (*τ*) are related to these parameters by *ρ* = m*/*Nq*^2^*τ* = 1/*qμN*. Explicit forms of the Drude model in terms of minority and majority carrier contributions for Si under dark condition and 1 sun illumination measurements are given in Supplementary Information Equations S1 and S2.

Optical anisotropy is introduced under non-zero applied magnetic field, and the Drude model is modified. The free charge (FC) carrier magneto-optic (MO) contribution to the frequency dependent dielectric tensor ***ε***(*ω*) is obtained from the equation of motion for a free charge carrier with a charge *q*, a scaled (unitless) effective mass tensor ***m***, and an frequency-independent scattering tensor ***γ***^17^:

2 $$\begin{array}{cc}{{\boldsymbol{\varepsilon }}}^{{\rm{FC}} \mbox{-} {\rm{MO}}}(\omega )={\boldsymbol{I}}+{{\boldsymbol{\omega }}}_{{\rm{p}}}^{2}\times  & {[-{\omega }^{2}{\boldsymbol{I}}-i\omega {\boldsymbol{\gamma }}+i\omega (\begin{array}{ccc}0 & {b}_{3} & -{b}_{2}\\ -{b}_{3} & 0 & {b}_{1}\\ {b}_{2} & -{b}_{1} & 0\end{array}){{\boldsymbol{\omega }}}_{c}]}^{-1},\end{array}$$

where, ***I***, ***B*** = *B* (*b*_1_, *b*_2_, *b*_3_), ***ω***_**p**_^2^ = (*Nq*^2^/*ε*_0_*m*_*e*_) ***m***^−1^, ***ω***_**c**_ = (*qB*/*m*_*e*_) ***m***^−1^, *ε*_0_, and *m*_*e*_ are the identity matrix, external magnetic field vector, plasma frequency tensor, cyclotron frequency tensor, vacuum permittivity, and electron mass, respectively. The *b*_1_, *b*_2_, and *b*_3_ matrix elements are the direction cosines of *B*. The non-zero differences in the magneto-optic Mueller matrix elements for +**B** and −**B** are related to free carrier motion generated by the Lorentz force. Analysis of Mueller matrix spectra enable determination of ***ω***_***p***_ and ***γ*** related to *N*/m* and m*μ. Fitting the difference in Mueller matrix elements, which correspond to the specific tensor elements of ***ω***_***c***_^17^, enables extraction of *N*, m*, and *μ* independently. Although the effective mass tensor elements of Si are complicated with six pockets in the Fermi surface and two effective mass parameters^21^, the analysis described here is simplified and a single conductivity effective mass for each Si layer is assumed^35^.

Bulk wafer thickness, air gap thickness, and optical properties for each of the active layers in the cell are extracted in the form of layer thicknesses and spectra in *ε*, respectively, by fitting a parameterized model to measured Mueller matrix spectra and Mueller matrix difference spectra collected at all three angles of incidence using a least square regression analysis that minimizes an unweighted error function (*σ*)^36^. The errors reported on model fit parameters are derived from a combination of the statistical standard 90% confidence limit and the overall error function quantifying the closeness of model fit to measurement. Consequently, these errors are best interpreted as a measure of model sensitivity to each parameter in the context of all other fit parameters.

## Results

**Figure 2:**
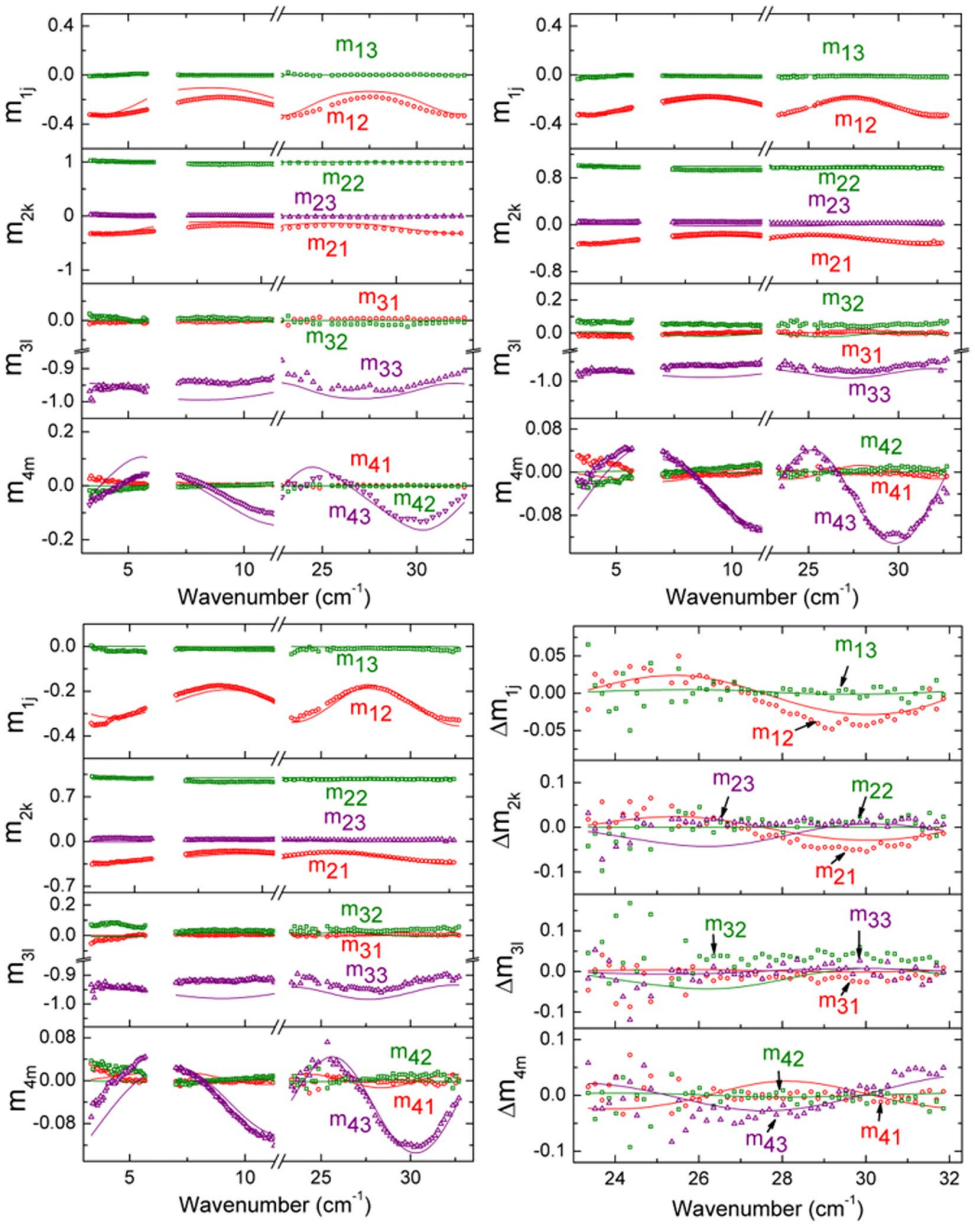
Experimental (symbols) spectra measured at 0 T (top-left), +1.48 T (top-right), −1.48 T (bottom-left) and difference spectra measured at ±1.48 T (bottom-right) with their corresponding calculated models (solid lines) of m_12_, m_13_, m_21_, m_22_, m_23_, m_31_, m_32_, m_33_, m_41_, m_42_, and m_43_ normalized Mueller matrix elements for the Al-BSF Si wafer solar cell at 50° angle of incidence under dark condition measurement. The unweighted error function for this fit is *σ* = 4.33 × 10^−2^.

**Figure 3:**
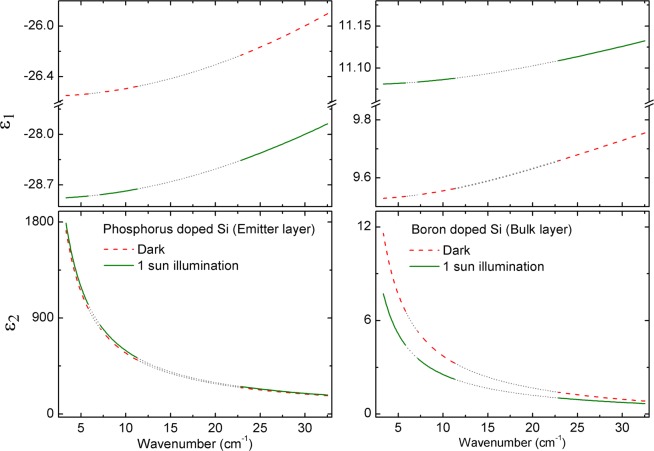
Complex dielectric function, *ε* = *ε*_1_ + i*ε*_2_, spectra for the phosphorus doped n-type Si emitter layer and boron doped p-type Si bulk wafer obtained from optical Hall effect measurements of the Al-BSF Si wafer solar cell under dark condition and 1 sun illumination (AM1.5 solar irradiance spectrum) measurements. Short dotted grey lines indicate gaps in the measured spectral range with the parametric expression for spectra in *ε* extrapolated over those regions.

The structural model consists of a 20 μm aluminum layer/181 ± 4 μm p-type boron doped Si wafer/1 μm n-type phosphorus doped Si emitter layer/75 nm SiN_x_ layer/air ambient, as shown in Fig. 1. The in-plane pyramidal texture feature size is ~4–5 μm^31^ and much smaller than the wavelengths of the probing THz beam; as a result, the surface is treated as specular for these measurement wavelengths. The bus bar front electrical contacts are avoided by the probing beam, and the metal finger bars occupy ~7% of the surface area of the probing beam spot. Contributions from these finger bars are not included in the optical model as the majority of the beam spot samples the surface of the Si solar cell. A discrete interface between the n-type Si emitter and the p-type Si wafer is assumed for simplicity. The p-type Si wafer thickness is a fit parameter, while all other thicknesses are fixed at nominal values from literature, noted in Fig. 1^31,32^. A similar structural-optical model is used in the presence of magnetic fields; however, a finite thickness air gap is included between the sample and the magnet. Spectra in *ε* for the active n- and p-type Si are extracted by fitting measured Mueller matrix and Mueller matrix difference spectra using a parameterized model. The three sets of data collected under dark condition measurement at 0 and ±1.48 T applied magnetic fields in spectral range windows from 3 to 34 cm^−1^ are fitted for this sample simultaneously with difference in Mueller matrix spectra [i.e. *Δm*_*ij*_ = *m*_*ij*_(+*B*) − *m*_*ij*_(−*B*)] from 23 to 32 cm^−1^. Figure 2 shows the example experimental Mueller matrix spectra collected at 50° under the dark condition from 3 to 34 cm^−1^ at 0, +1.48, −1.48 T; experimental Mueller matrix difference spectra from 23 to 32 cm^−1^; and corresponding best fit models. Contributions to THz range spectra in *ε* for the phosphorus doped n-type Si emitter layer of the cell include a constant additive term to *ε*_1_ (*ε*_∞_ = 1) and a Drude expression^34^ describing free carrier absorption, with the real and imaginary parts of the spectra in *ε* shown in Fig. 3. Similarly, the spectra in *ε* for the boron doped p-type Si wafer are parameterized by *ε*_*∞*_ = 10.3 ± 0.4 and a Drude expression^34^ describing free carrier absorption with spectra in *ε* also shown in Fig. 3. For the more heavily doped n-type Si emitter the Drude contribution dominates the shape of *ε* so sensitivity to *ε*_∞_ is lost and its value fixed at unity, whereas for the more lightly doped p-type Si wafer the Drude contribution is less dominant and sensitivity to *ε*_∞_ is retained. Optical response of the permanent magnet consists of the Drude expression for highly reflective gold as described in the approach of Knight *et al*.^19^ for optical Hall effect measurements. The effects due to the 75 nm SiN_x_ coating are negligible in this long wavelength spectral range. Spectra in *ε* for the aluminum back contact are represented by the parametric expression described by Subedi *et al*.^37^.

**Figure 4:**
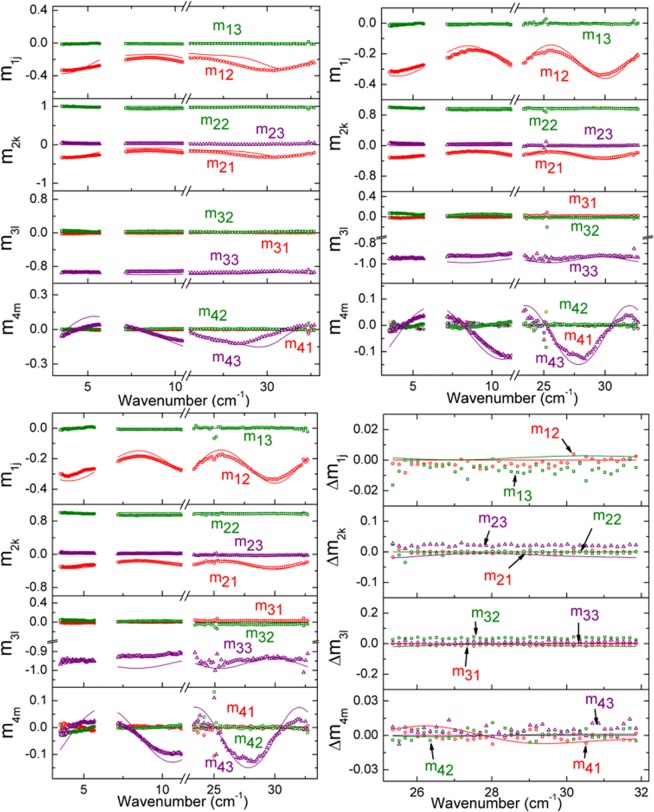
Experimental spectra (symbols) measured at 0 T (top-left), +1.48 T (top-right), and −1.48 T (bottom-left) and difference spectra measured at ±1.48 T (bottom-right) with their corresponding calculated models (solid lines) of the normalized Mueller matrix elements m_12_, m_13_, m_21_, m_22_, m_23_, m_31_, m_32_, m_33_, m_41_, m_42_, and m_43_ for the Al-BSF Si wafer solar cell at 50° angle of incidence under 1 sun illumination (AM1.5 solar irradiance spectrum). The unweighted error function for this fit is *σ* = 4.29 × 10^−2^.

The parameters describing majority carrier free carrier absorption for the n-type emitter and p-type Si wafer obtained from measurements collected under dark conditions are summarized in Table 1. Optical Hall effect measurements of the same solar cell are performed under 1 sun illumination and a similar data analysis approach is applied. Figure 4 shows example experimental Mueller matrix spectra collected at 50° under 1 sun illumination from 3 to 34 cm^−1^ at 0, +1.48, −1.48 T; Mueller matrix difference spectra from 25 to 32 cm^−1^; and corresponding best fit models. The wafer thickness is fixed to 181 μm from analysis of optical Hall effect measurements collected under dark conditions. Under 1 sun illumination, spectra in *ε* for the n-type emitter are parameterized using *ε*_∞_ = 1 and two Drude expressions separately representing contributions to free carrier absorption from majority carrier electrons and minority carrier holes, shown in Fig. 3. The hole contribution to free carrier absorption is due to photogenerated carriers under 1 sun illumination. The electron contribution consists of an equal concentration of photogenerated carriers added to the carriers from phosphorus doping. Similarly, spectra in *ε* for the p-type bulk wafer are parameterized by *ε*_∞_ = 11.41 ± 0.02 with Drude expressions for photogenerated minority carrier electrons and a combination of majority carrier holes from boron doping and photogeneration, also shown in Fig. 3. As in the case of the dark condition measurement analysis, the dominance of the Drude contributions to *ε* causes a loss of sensitivity to *ε*_∞_ for the heavily doped n-type emitter while it is retained for the lightly doped p-type wafer. The majority and photogenerated minority carrier transport properties for the emitter and bulk Si layers of the cell are summarized in Table 1. In the model applied to analysis of the 1 sun illuminated measurements, the respective impurity doping carrier concentrations and conductivity effective masses are fixed to values obtained from the dark condition measurements. Photogenerated electron and hole concentrations are equal within each layer. Electron and hole *μ* in each layer are allowed to vary independently to account for additional recombination processes expected under illumination.

**Table 1:** Dark and light carrier transport properties for Si phosphorus-doped n-type emitter and boron-doped p-type wafer in the Al-BSF solar cell.

Layers	Conditions	Transport Property	
Phosphorus-doped n-type Si emitter layer	Dark	Electron mobility *μ* (cm^2^/V s)	166 ± 6
		Electron carrier concentration *N* (cm^−3^)	(3.6 ± 0.1) × 10^18^
		Electron effective mass m* (×*m*_*e*_)	0.28 ± 0.03
		Resistivity *ρ* (Ωcm)	0.01
		Scattering time *τ* (fs)	26
	Light (1 sun illumination)	Photogenerated electron and hole concentration *Δn* or *Δp* (cm^−3^)	(7.8 ± 0.2) × 10^16^
		Electron mobility *μ*_*e*_ (cm^2^/Vs)	162 ± 5
		Hole mobility *μ*_*h*_ (cm^2^/Vs)	331 ± 191
Boron-doped p-type Si wafer	Dark	Hole mobility *μ* (cm^2^/Vs)	532 ± 12
		Hole carrier concentration *N* (cm^−3^)	(7.6 ± 0.1) × 10^15^
		Hole effective mass m* (×*m*_*e*_)	0.36 ± 0.02
		Resistivity *ρ* (Ωcm)	1.5
		Scattering time *τ* (fs)	109
	Light (1 sun illumination)	Photogenerated hole and electron concentration *Δp* or *Δn* (cm^−3^)	(2.2 ± 0.2) × 10^14^
		Hole mobility *μ*_*h*_ (cm^2^/Vs)	323 ± 32
		Electron mobility *μ*_*e*_ (cm^2^/Vs)	766 ± 331

## Discussion

### Optically deduced transport properties

These optically deduced transport properties are similar to those reported in literature. Carrier concentrations for the n-type phosphorus doped Si emitter and p-type boron doped Si bulk are comparable to the expected values of *N* ~10^18^ and ~10^15^ cm^−3^, respectively, for Si found in commercially produced cells^32,38^. The mobilities extracted for n- and p-type Si are consistent with literature values of 133 and 429 cm^2^/Vs, respectively, for the corresponding *N*^39^. m* for both phosphorus doped n-type and boron doped p-type Si agree with reported values of 0.26 and 0.39*m*_*e*_, respectively^40,41^. *ρ* and *τ* determined from the Drude equation show good agreement with reported resistivities of 0.01 and 1.9 Ω cm and scattering times of 24 and 95 fs, for n- and p-type Si, respectively, with the corresponding *N*^39–41^. Overall, all three majority carrier transport parameters (*N*, *μ*, m*) evaluated from the Drude model are reliably obtained for the n-type emitter and p-type bulk Si of the Al-BSF solar cell by the optical Hall effect measurements.

Electron and hole *μ* in each layer can vary independently to account for additional recombination processes expected under illumination. Under illumination, radiative^42–44^, Auger^9,45,46^, and Shockley-Read-Hall^47^ recombination take place along with photogeneration of carriers. Photogenerated hole or electron concentration in the phosphorus doped n-type Si emitter is determined to be (7.8 ± 0.2) × 10^16^ cm^−3^, which is ~2% of the impurity dopant or dark carrier concentration. Majority carrier electron mobility under 1 sun illumination is 162 ± 5 cm^2^/Vs, which is comparable to that obtained from dark measurements. Minority carrier hole mobility is 331 ± 191 cm^2^/Vs. Sproul *et al*., Swirhun *et al*., Alamo *et al*., and Law *et al*.^6,10,11,13^ reported that minority carrier hole mobility in n-type Si is generally higher than that of majority carrier hole mobility in p-type Si when impurity doped to similar levels; the latter hole mobility ranges from 450 to 80 cm^2^/V s for carrier concentrations in the range from ~1 × 10^16^ to 4 × 10^18^ cm^−3^. This difference is dopant concentration dependent for Si, which is at a minimum for low dopant concentration and increases with increasing dopant concentration. Here the minority carrier hole mobility in phosphorus doped Si is approximately double that of the majority carrier electron mobility, which is within expectations for Si with this impurity doping level^6,7,16,39^.

The photogenerated electron and hole concentrations in the boron doped p-type Si bulk layer are (2.2 ± 0.2) × 10^14^ cm^−3^, which is ~3% of the boron impurity dopant or dark carrier concentration. Hole mobility under 1 sun illumination is 323 ± 32 cm^2^/Vs, which is relatively lower than the measured dark value, but still comparable to literature expectations^39^. Minority carrier electron mobility is determined to be 766 ± 331 cm^2^/Vs. Majority carrier electron mobility in 1.2 × 10^16^ cm^−3^ carrier concentration phosphorus impurity doped n-type Si is reported at 1093 cm^2^/Vs^39^. Neuhaus *et al*. and Rougieux *et al*. have reported that the sum of minority and majority mobility approaches 1500 cm^2^/Vs in ~1 Ω cm resistivity p-type Si with 10^16^ cm^−3^ impurity dopant concentration^7,16^. Swirhun *et al*. have reported minority electron mobility of 700–800 cm^2^/Vs in material with an acceptor concentration of 10^17^ cm^−3^ ^10^. Sproul *et al*. have also reported minority carrier electron mobility of ∼ 1000 cm^2^/Vs for 10^16^ cm^−3^ impurity carrier concentration boron doped Si^6^. The minority carrier mobility from optical Hall effect is slightly lower than these values but within expectation in consideration of the confidence limits^7,16^. Additionally, optical Hall effect measurements reported here are under 1 sun illumination in comparison to other techniques such as photoconductance decay^6^, quasi-steady-state photoconductance + open-circuit voltage^7^, and transient + quasi-steady-state photoconductance measurements^16^. In photoconductance decay, charge carrier generation is achieved optically by a short laser pulse, and carrier decay is measured by changes in microwave reflectance indicative of the rate at which carriers recombine after a short excitation. Quasi-steady-state photoconductance decay measures the balance between generation and recombination when quasi-steady-state illumination is achieved^6,16^. For quasi-steady-state photoconductance and *V*_*oc*_ techniques, both are measured under flash lamp illumination of different intensities^7^. Variation in values measured by these different techniques may arise from the different illumination levels used. Since these optical Hall effect measurements are performed under 1 sun illumination on a complete PV device, the transport properties measured by this method may more accurately represent the electrical transport properties of Si in this type of solar cell under operating conditions.

The optical and structural model assumes uniform carrier concentrations throughout the thicknesses of the n-type emitter and p-type bulk wafer for simplicity, although Al-BSF Si solar cells are more complicated with a n-type doping concentration gradient following a Gaussian or Gauss error function profile^48^ and a depletion region present. Hofmann *et al*. studied p-p+ Si homojunctions using THz to mid infrared frequency range ellipsometry and deduced a carrier concentration profile utilizing relationships between hole concentration and mobility^35^. Analysis of the optical Hall effect measurements here yield majority and minority average carrier concentrations within each doped Si region with carrier mobilities as independent fit parameters. The analysis did not provide sensitivity to the n-type emitter carrier concentration profile or the characteristics of the depletion region. Sensitivity to these characteristics may be lost due to the structure of this commercial Al-BSF device and overlapping optical effects of the n-type carrier concentration profile and depletion region transport properties occurring near the top of the Si junction. Although the simplified model of a discrete n-type emitter and p-type bulk Si interface is applied in analysis of the optical Hall effect data, the transport properties determined are used to calculate reasonable minority carrier lifetimes and diffusion lengths as well as to simulate solar cell performance parameters consistent with experiment.

### Minority carrier lifetime and diffusion length

Different recombination mechanisms are used to determine the lifetime and diffusion length of the minority charge carriers in the emitter and bulk components of the solar cell. Minority carrier recombination lifetime (*τ*_r_) is determined by *Δn*/*U*_*r*_ or *Δp*/*U*_*r*_, where *Δn* or *Δp* represents the photogenerated carriers and *U*_*r*_ is the recombination rate. All equations are provided in the Supplementary Information. The minority carrier diffusion length is then calculated by (*Dτ*_r_)^1/2^, where *D* is diffusivity given by the Einstein-Smoluchowski relation (*D* = *μkT*/*q*), *μ* is minority carrier mobility, *k* is Boltzmann’s constant, *T* is ambient temperature and fixed at 300 K, and *q* is electron charge. From the mobility values in Table 1, the minority carrier diffusivities of the emitter and bulk are determined as 8 and 19.8 cm^2^ s^−1^, respectively. Using the dark electron concentration of 3.6 × 10^18^ cm^−3^ and the photogenerated hole concentration of 7.8 × 10^16^ cm^−3^, effective hole lifetimes associated with radiative, Auger, and Shockley-Read-Hall recombination mechanisms are calculated as presented in Table 2. For radiative recombination, lifetimes in the emitter are calculated to be 26, 56, and 67 μs using the parametric expressions reported by Schlangenotto *et al*.^42^, Trupke *et al*.^43^, and Nguyen *et al*.^44^, respectively. Similarly, for Auger recombination, lifetimes in the emitter are calculated to be 0.3 and 0.2 μs using the approach by Dziewior *et al*.^9^ and Kerr *et al*.^46^, respectively. The Shockley-Read-Hall lifetime is calculated to be 102 μs^47^. Thus, as shown in Table 2, the calculated lifetimes vary from 0.2 to 102 μs depending upon the recombination mechanism and model. Corresponding hole diffusion lengths are calculated to vary from 13 to 286 μm. As Si is an indirect band gap semiconductor, Auger and Shockley-Read-Hall recombination mechanisms dominate in these solar cells. Specifically, Auger recombination is dominant in the heavily doped emitter^9,45,46^, so the Auger recombination lifetimes^9,45^ are found to be consistent with lifetimes <1 μs commonly reported^13,49^. The diffusion lengths calculated from the Auger lifetimes are 13 and 15 μm, which are consistent with the 10 to 100 μm range reported in literature^13,49^.

**Table 2:** Minority carrier lifetime and diffusion length for phosphorus-doped n-type Si emitter and boron-doped p-type Si wafer in the Al-BSF solar cell assuming differnt recombination mechanisms and models.

Layers	Mechanism	Model	Lifetime (μs)	Diffusion length (μm)
Phosphorus-doped n-type Si emitter layer	Radiative	Schlangenotto *et al*.^42^	26	144
		Trupke *et al*.^43^	56	212
		Nguyen *et al*.^44^	67	231
	Auger	Dziewior *et al*.^9^	0.3	15
		Kerr *et al*.^46^	0.2	13
	Shockley-Read-Hall	Shockley *et al*.^47^	102	286
Boron-doped p-type Si wafer	Radiative	Schlangenotto *et al*.^42^	1.2 × 10^4^	5 × 10^3^
		Trupke *et al*.^43^	2.6 × 10^4^	7 × 10^3^
		Nguyen *et al*.^44^	3.2 × 10^4^	8 × 10^3^
	Auger	Dziewior *et al*.^9^	1.6 × 10^5^	2 × 10^4^
		Kerr *et al*.^46^	3.4 × 10^3^	3 × 10^3^
	Shockley-Read-Hall	Shockley *et al*.^47^	103	453

For the p-type Si bulk with a dark hole concentration of 7.6 × 10^15^ cm^−3^ and photogenerated electron concentration of 2.2 × 10^14^ cm^−3^, the electron lifetime in the bulk using different recombination mechanisms and models is found to range from 103 to 1.6 × 10^5^ μs^9,42–47^. Radiative recombination lifetimes in the bulk are calculated to be 1.2 × 10^4^, 2.6 × 10^4^, and 3.2 × 10^4^ μs using the parametric expressions reported by Schlangenotto *et al*.^42^, Trupke *et al*.^43^, and Nguyen *et al*.^44^, respectively. Similarly, Auger recombination lifetimes in the bulk are calculated to be 1.6 × 10^5^ and 3.4 × 10^3^ μs using the approaches by Dziewior *et al*.^9^ and Kerr *et al*.^46^, respectively. The Shockley-Read-Hall lifetime is calculated to be 103 μs. The corresponding electron diffusion lengths are calculated to range from 453 to 2 × 10^4^ μm. As the Shockley-Read-Hall recombination mechanism is dominant in the relatively lightly doped Si bulk wafer of the solar cell^47^, the lifetime calculated from this mechanism agrees well with ∼100 μs lifetimes reported^13,50^. The 453 μm diffusion length associated with Shockley-Read-Hall lifetime shown in Table 2 is consistent with the 100–1000 μm range reported in literature^13^. Overall, Auger and Shockley-Read-Hall recombination mechanisms are dominant in the emitter and bulk layers of the Si solar cells, respectively, and hence the lifetimes and diffusion lengths calculated from these mechanisms are found to be the most consistent with other reported lifetimes.

### PV Device performance parameters

The transport properties of the Si emitter and bulk wafer components of the solar cell directly impact PV device performance parameters. For an ideal diode, the dark current density^51^ is:

3 $${J}_{dark}(V)={J}_{o}({e}^{qV/kT}-1),$$

where, *J*_*o*_ = *qn*_*i*_^2^ [*D*_*n*_/*L*_*n*_*N*_*A*_ + *D*_*p*_/*L*_*p*_*N*_*D*_] = 1.1 × 10^−9^ mAcm^−2^, *q* is electron charge, *T* = 300 K here, and *n*_*i*_ = 1.08 × 10^10^ cm^−3^ is intrinsic carrier concentration^52,53^. *D*_*n*_ and *D*_*p*_ are diffusivities of the electron and hole minority carriers, *L*_*n*_ and *L*_*p*_ are the minority carrier electron and hole diffusion lengths, and *N*_*A*_ and *N*_*D*_ are acceptor and donor concentrations, respectively. Under illumination, the net current density^51^ is:

4 $$J={J}_{sc}-{J}_{dark}(V)$$

where *J*_*sc*_ is short circuit current density. The dark photocurrent density (*J*_*dark*_) is negligible compared to *J*_*sc*_ under 1 sun illumination. *J*_*sc*_ is measured to be 37.0 ± 0.3 mAcm^−2^ for the solar cell of Fig. 1. The calculated *V*_*oc*_ = *kT*/*q* ln(*J*_sc_/*J*_o_ + 1) using measured *J*_*sc*_ is 0.626 V. The normalized open circuit voltage *v*_*oc*_ [*v*_*oc*_ = *V*_*oc*_/(*kT*/*q*)] is 24.3. The idealized fill factor [*FF* = {*v*_*oc*_ − ln(*v*_*oc*_ + 0.72)}/(*v*_*oc*_ + 1)] is calculated to be 0.833. Resultant simulated PV device efficiency (*η* = *V*_*oc*_*J*_*sc*_*FF*/100 mWcm^−2^) is 19.2%. These *V*_*oc*_, *FF*, and *η* values calculated from the transport properties obtained from optical Hall effect using measured *J*_*sc*_ are in good agreement with other measured device performance parameters *V*_*oc*_ = 0.627 ± 0.001 V, *FF* = 0.80 ± 0.02, and *η* = 18.5 ± 0.1%. Thus, simulated *V*_*oc*_ based upon input from this study is within error of the measured value. The calculation of *FF* = 0.833 assumes infinite shunt resistance and negligible series resistance and is considered as an idealized limit, with the measured *FF* observed to be lower at 0.80. From *J-V* measurements, series and shunt resistances of the solar cell are determined to be 0.62 and 5.5 × 10^4^ Ω cm^2^, respectively. Using the approach described by Mette *et al*.^54^, the *FF* accounting for series resistance is calculated to be 0.80. As the shunt resistance is very high, measured *V*_*oc*_ and *J*_*sc*_ do not substantially deviate from idealized simulations^55^, indicating that the series resistance contribution of 0.62 Ω cm^2^ is the likely cause of the reduction in expected *FF* from 0.833 to 0.80^54,56^. After accounting for the reduction in *FF* due to series resistance, the resultant calculated efficiency is 18.4%, which matches the measured efficiency.

More detailed simulations of solar cell performance are performed using PC1D^57^. Using PC1D, electron and hole transport equations are solved for carriers in the complete Si solar cell described in Fig. 1 to determine *J-V* characteristics. Input parameters include those derived from optical Hall effect measurements reported in Tables 1 and 2 when applicable with all input parameters provided in the Supplementary Information. In PC1D the carrier concentration profile of the n-type emitter is manipulated to account for non-uniform carrier concentration with depth. Doping carrier concentrations simulated include a uniform distribution represented by the average carrier concentration reported in Table 1, an exponential profile, a Gaussian profile, and a Gauss error function profile. For the last three, all profiles are defined by the maximum n-type carrier concentration in the layer, which is constrained by the shape of the profile so that numerical integration for determining the average carrier concentration corresponds to that reported in Table 1. The surface recombination velocity (*S*_*p*_) at the doped n-type front emitter is determined to be of *S*_*p*_ = 2.5 × 10^4^ cm/s in order to best match the experimental *J-V* characteristics, as reported in Supplemental Information Table S1. All other parameters are constrained to values reported in literature. PC1D simulation results incorporating uniform and Gauss error function carrier concentration profiles in the n-type emitter most closely match experimental values with simulated *V*_*oc*_ = 0.621 and 0.625 V, *J*_*SC*_ = 37.3 and 37.2 mA/cm^2^, *FF* = 0.799 and 0.800, and resulting *η* = 18.5 and 18.6%, for uniform distribution and Gauss error function profiles, respectively. Although both simulation results match experimental values, the carrier concentration profile is unlikely to be completely uniform. Based on the similarity of the results from these two simulations, it is possible that the simulated n-type emitter carrier concentration profile is more complicated than either a uniform distribution or Gauss error function profile individually, although the precise profile is not known for this commercial Al-BSF Si wafer solar cell. Average majority and minority carrier concentrations in the n-type emitter and p-type bulk wafer are obtained from the simplified model consisting of discrete layers of uniform carrier concentration used in analysis of optical Hall effect measurements. The agreement between the PC1D simulated and experimental *J-V* characteristics supports the average carrier concentration values obtained from the optical Hall effect characterization of this device. These optical Hall effect input parameters may serve as further constraints in determining solar cell characteristics via device modeling.

An evaluation of all these results leads to the conclusion that a combined analysis of optical Hall effect measurements collected under dark and illuminated conditions is sensitive to majority carrier transport properties from impurity doping and minority carrier transport properties from carrier photogeneration under 1 sun illumination. In particular, *N*, *μ*, and m* are obtained for both the phosphorus doped n-type Si emitter and the boron doped p-type Si bulk of an Al-BSF solar cell from dark measurements. Based on information gained from analysis of the dark measurements, minority carrier transport parameters in both the n- and p-type Si components of the Al-BSF solar cell are deduced from analysis of measurements collected under 1 sun illumination. Consistency between calculated device performance parameters using transport properties via optical Hall effect and measured values demonstrates the reliability of this novel technique for transport properties measurement even in a complicated structure such as an Al-BSF Si solar cell.

## Conclusion

As optical Hall effect measurements are non-contacting and sensitive to the optical response of multiple layers in a sample stack, it has the potential to determine transport parameters of the individual layers in multilayer PV device structures, which may be inaccessible by direct measurements such as the electrical Hall effect. Majority and minority carrier transport properties of Si active layers within an Al-BSF solar cell are deduced from the optical Hall effect measurements performed under nominally dark conditions and under 1 sun illumination. The minority carrier transport parameters, *Δp* or *Δn* and *μ*_*h*_ or *μ*_*e*_, as well as the majority carrier transport parameters *N*, *μ*, and m*, are determined for the phosphorus doped n-type Si emitter and boron doped p-type Si wafer in an Al-BSF configuration solar cell with pyramidal front surface texturing, a relatively common commercially produced device structure. The majority carrier transport properties extracted here agree well with those reported in the literature. Minority carrier mobilities and concentrations of the active layers of the cell are also within expectations. The calculated minority carrier effective lifetime and diffusion length from *Δp* or *Δn* and *N* for each layer also show good agreement with reported values for similar dopant densities, which demonstrate the validity of this technique for determination of minority carrier transport properties. Calculated PV device performance parameters from measured transport properties via optical Hall effect agree well with directly measured values, further validating this method for reliably obtaining majority and minority carrier transport properties. Using this non-contacting optical technique both majority and minority carrier transport properties are measured within a complete solar cell, providing a pathway to improved understanding of fundamental electrical properties of materials when integrated into opto-electronic devices.

### Index terms

Minority carriers, Optical Hall effect, Transport parameters, Al-BSF Si wafer solar cell, THz ellipsometry.

## Supplementary information

Supporting Information
