# Supplementary material for: Photogenerated Carrier Transport Properties in Silicon Photovoltaics
Source: Sci Rep. 2019 Dec 12;9:19015. doi: 10.1038/s41598-019-55173-z (PMC6908689; doi:10.1038/s41598-019-55173-z)
Supplement: Supplementary file 1 — Supporting Information [file 41598_2019_55173_MOESM1_ESM.pdf]

SUPPLEMENTARY INFORMATION FOR:

PHOTOGENERAGED CARRIER TRANSPORT PROPERTIES IN SILICON PHOTOVOLTAICS

Prakash Uprety, Indra Subedi, Maxwell M. Junda, Robert W. Collins, and Nikolas J. Podraza

Wright Center for Photovoltaics Innovation and Commercialization &

Department of Physics & Astronomy,

University of Toledo, Toledo, OH 43606, USA

1. COMPLEX DIELECTRIC FUNCTION MODEL UNDER DARK AND 1 SUN ILLUMINATION CONDITIONS

The complex dielectric function ($\varepsilon = \varepsilon_1 + i\varepsilon_2$) model for Si under 1 sun illumination includes contributions from the constant additive term to the real part of ε (ε_1) denoted as ε_∞, a Drude model accounting for majority carriers, and another Drude model accounting for minority carriers. It is described as:

$$\varepsilon_{illuminated}\left(\omega\right)=\varepsilon_{\infty}-\frac{q^{2}(N_{majority,dark}+\Delta N_{majority,photogenerated})\mu_{majority}}{\varepsilon_{0}\left(m^{*}_{majority}\mu_{majority}\omega^{2}+iq\omega\right)}-\frac{q^{2}\Delta N_{minority,photogenerated}\ \mu_{minority}}{\varepsilon_{0}\left(m^{*}_{minority}\mu_{minority}\omega^{2}+iq\omega\right)}$$

(Equation S1)

where q is the fundamental electron charge, ω is the frequency of probing light, ε_0 is the permittivity of free space, m^* are the conductivity effective masses, μ are the carrier mobilities, $N_{majority,dark}$ is the carrier concentration of the majority carriers measured under dark conditions, and $\Delta N_{majority,photogenerated}$ = $\Delta N_{minority,photogenerated}$ are the equal contributions from photogenerated majority and minority carriers. The transport parameters of both minority and majority carriers for each layer are determined from the parameterization of free carrier absorption observed under illumination using these two Drude oscillators.

In the dark condition measurement, the majority carrier concentration is much greater than that of the minority carrier concentration. The model simplifies to:

$$\varepsilon_{dark}(\omega) = \varepsilon_{\infty} - \frac{q^2 N_{majority,dark}\, \mu_{majority}}{\varepsilon_0 \left(m^*_{majority} \mu_{majority} \omega^2 + iq\omega \right)}$$ (Equation S2).

2. MINORITY CARRIER LIFETIME CALCULATION

Minority recombination lifetime for each mechanism is given by:

$\tau_r = \Delta n / U_r$ or $\Delta p / U_r$ (Equation S3)

where Δn or Δp represents the photogenerated carrier concentrations and U_r represents the mechanism recombination rate. Dopant and photogenerated carrier concentrations are reported in Table 1. For the n-type Si emitter, dopant electron concentration is $n = 3.6 \times 10^{18}$ cm^{-3} and photogenerated carrier concentrations are $\Delta n = \Delta p = 7.6 \times 10^{16}$ cm^{-3}. For the p-type Si wafer, dopant hole concentration is $p = 7.6 \times 10^{15}$ cm^{-3} and photogenerated carrier concentrations are $\Delta n = \Delta p = 2.2 \times 10^{14}$ cm^{-3}. For Si, the effective intrinsic carrier concentration is $n_{ieff} = 1.1 \times 10^{10}$ cm^{-3}. For the phosphorus doped n-type Si emitter, the equilibrium electron (n_0) and hole (p_0) concentrations are $n_0 = 2.8 \times 10^{18}$ cm^{-3} and $p_0 = 144$ cm^{-3}. For the boron doped p-type Si wafer, $p_0 = 7.56 \times 10^{15}$ and $n_0 = 1.42 \times 10^{4}$ cm^{-3}. Temperature is $T = 300$ K.

2.1. RADIATIVE RECOMBINATION

Radiative recombination rates are calculated using the parametric expressions reported by Schlangenotto et al. [42, SI A], Trupke et al. [43, SI A], and Nguyen et al. [44, SI A], respectively. These expressions are:

Schlangenotto et al. [42, SI A]:

$$U_{rad} = \left(1.0391 \times 10^{-14} + 5.213 \times 10^{-12} e^{-T/29.86338}\right)\left(pn - n_{ieff}^2\right)$$ (Equation S4)

Trupke et al. [43, Si A]:

$$U_{rad} = \left(4.6767\times10^{-7}\big/ T^{3.60927} + 4.4111\times10^{-15}\right)\left(pn - n_{ieff}^2\right) \qquad \text{(Equation S5)}$$

Nguyen et al. [44, SI A]:

$$U_{rad} = 10^{\left(-176.98+2.68812T-0.018137T^2+6.56769\times10^{-5}T^3-1.21382\times10^{-7}T^4+8.99086\times10^{-11}T^5\right)}.\left(pn\big/ n_{ieff}^2 - 1\right) \qquad \text{(Equation S6)}$$

2.2. AUGER RECOMBINATION

Auger recombination rates are calculated using the parametric expressions reported by Dziewior et al. [9, SI A], and Kerr et al. [46, SI A].

Dziewior et al. [9, SI A]:

$$U_{Aug} = \left(1.0391\times10^{-14} + 5.213\times10^{-12} e^{-T/29.86338}\right)\left(pn - n_{ieff}^2\right) \qquad \text{(Equation S7)}$$

Kerr et al. [46, SI A]:

$$U_{Aug} = np\left(1.8\times10^{-24} n_0^{0.65} + 6\times10^{-25} p_0^{0.65} + 3\times10^{-27} \Delta n^{0.8}\right) \qquad \text{(Equation S8)}$$

2.3. SHOCKLEY-READ-HALL RECOMBINATION

Shockley-Read-Hall (SRH) recombination rate is calculated using [47, 50, SI A]:

$$U_{SRH} = \frac{pn - n_{ieff}^2}{\tau_{no}(p + p_1) + \tau_{po}(n + n_1)} \qquad \text{(Equation S9)}$$

$$\tau_{po} = \frac{1}{v_{thh}\sigma_p N_t}$$
$$\tau_{no} = \frac{1}{v_{the}\sigma_n N_t}$$
(Equation S10)

$$n_1 = n_{ieff}\exp(\frac{E_t - E_i}{kT})$$
$$p_1 = n_{ieff}\exp(\frac{E_i - E_t}{kT})$$
(Equation S11)

where σ_n and σ_p are the capture cross sections of electrons and holes, $v_{th\,e}$ and $v_{th\,h}$ are the thermal velocities of electrons and holes, N_t are concentration and E_t are defect state concentration and the energy, and E_i is the intrinsic Fermi energy. $N_t << p, n$. Electron and hole lifetimes are assumed to be $\tau_{no} = \tau_{po} =$ 100 μs [SI A] and for simplicity $E_t - E_i = 0$.

3. PC1D SOLAR CELL SIMULATION INPUT PARAMETERS

Device area: 100 cm^2

Front surface texture depth: 3.5 μm [37]

No surface charges

Exterior Front Reflectance: Included the reflectance of bare Al-BSF from PV Lighthouse [37].

No Exterior Rear Reflectance

Internal optical reflectance enabled

Front surface optically rough

Emitter contact enabled

Base contact: 0.0062 Ω

Internal conductor: 1.8×10^{-3} S

Thickness: 180 μm

Material modified from si.mat : Modified to include new refractive index and absorption coefficient from reference. [SI B]

Carrier mobilities from internal model (Varying mobility)

Dielectric constant: 11.9

Band gap: 1.124 eV

Intrinsic carrier concentration at 300 K: 1.08×10^{10} cm^{-3}

Refractive index and absorption coefficient from Ref. [SI B]

Free carrier absorption enabled

p-type background doping: 7.6×10^{15} cm^{-3}

1st front n-type diffusion cases (i-iv):

(i) 3.6×10^{18} cm^{-3} uniform carrier concentration

(ii) 5.7×10^{18} cm^{-3} peak carrier concentration for an exponential profile; determined assuming an average 3.6×10^{18} cm^{-3} carrier concentration

(iii) 4.9×10^{18} cm^{-3} peak carrier concentration for a Gaussian profile; determined assuming an average 3.6×10^{18} cm^{-3} carrier concentration

(iv) 7.5×10^{18} cm^{-3} peak carrier concentration for a Gauss error function profile; determined assuming an average 3.6×10^{18} cm^{-3} carrier concentration

Depth factor: 1 μm

No 2nd front diffusion

No rear diffusion

Bulk recombination: $\tau_n = \tau_p = 100$ μs [SI A]

Front-surface recom.: S model, $S_n = S_p = 25{,}000$ cm/s

Rear-surface recom.: S model, $S_n = S_p = 100$ cm/s [SI C]

EXCITATION:

Excitation from one-sun.exc

Excitation mode: Transient, 16 timesteps

Temperature: 300 K

Base circuit: Sweep from -0.8 to 0.8 V

Collector circuit: Zero

Primary light source enabled

Constant intensity: 0.1 W cm^{-2}

Spectrum from am15g.spc

Secondary light source disabled

TABLE S1
J-V PERFORMANCE COMAPRISION BETWEEN PC1D SIMULATIONS WITH DIFFERENT DOPING CONCENTRATION PROFILES AND EXPERIMENT

**Parameters**	**Experiment**	**Uniform**	**Gauss Error Function**	**Exponential**	**Gaussian**
V_{oc} (V)	0.627	0.621	0.625	0.625	0.624
J_{sc} (mA/cm^2)	37.0	37.3	37.2	32.5	35.9
FF	0.796	0.799	0.800	0.803	0.800
Efficiency (%)	18.5	18.5	18.6	16.3	17.9

References

[SI A] https://www2.pvlighthouse.com.au/calculators/Recombination%20calculator/Recombination%20 calculator.aspx.

[SI B] Schinke, C. *et al.* Uncertainty analysis for the coefficient of band-to-band absorption of crystalline silicon. *AIP Advances* **5**, 067168, 2015

[SI C] Cai X, Zhou X, Liu Z, Jiang F, Yu Q, An in-depth analysis of the silicon solar cell key parameters optimal magnitudes using PC1D simulations, *Optik* ***164***, 105-113, 2018.
